# Maternal stress-associated cortisol stimulation may protect embryos from cortisol excess in zebrafish

**DOI:** 10.1098/rsos.160032

**Published:** 2016-02-24

**Authors:** Erin Faught, Carol Best, Mathilakath M. Vijayan

**Affiliations:** Department of Biological Sciences, University of Calgary, 2500 University Drive NW, Calgary, Alberta, Canada T2N 1N4

**Keywords:** fish, cortisol, stress response, early development, *11βHSD2*

## Abstract

Abnormal embryo cortisol level causes developmental defects and poor survival in zebrafish (*Danio rerio*). However, no study has demonstrated that maternal stress leads to higher embryo cortisol content in zebrafish. We tested the hypothesis that maternal stress-associated elevation in cortisol levels increases embryo cortisol content in this asynchronous breeder. Zebrafish mothers were fed cortisol-spiked food for 5 days, to mimic maternal stress, followed by daily breeding for 10 days to monitor temporal embryo cortisol content. Cortisol treatment increased mean embryo yield, but the daily fecundity was variable among the groups. Embryo cortisol content was variable in both groups over a 10-day period. A transient elevation in cortisol levels was observed in the embryos from cortisol-fed mothers only on day 3, but not on subsequent days. We tested whether excess cortisol stimulates *11βHSD2* expression in ovarian follicles as a means to regulate embryo cortisol deposition. Cortisol treatment *in vitro* increased *11β* *HSD2* levels sevenfold, and this expression was regulated by actinomycin D and cycloheximide suggesting tight regulation of cortisol levels in the ovarian follicles. We hypothesize that cortisol-induced upregulation of *11βHSD2* activity in the ovarian follicles is a mechanism restricting excess cortisol incorporation into the eggs during maternal stress.

## 1. Introduction

Plasma cortisol, the primary corticosteroid in teleosts, levels increase in response to stressor exposure in fishes [1]. This involves the activation of hypothalamus–pituitary–interrenal (HPI) axis, including the release of corticotropin releasing factor from the hypothalamus, which stimulates the anterior pituitary to secrete adrenocorticotropic hormone (ACTH) [2,3]. ACTH acts on the interrenal tissue (analogous to the adrenal gland in mammals) to release cortisol into the circulation. Cortisol acts on target tissues to bring about physiological changes essential to regain homeostasis, and this is mediated by non-genomic and genomic signalling, including glucocorticoid receptor and mineralocorticoid receptor activation [4,5]. Target tissue regulation of cortisol action also involves the inactivation of this steroid to its inactive metabolite cortisone by the enzyme 11*β*-hydroxysteroid dehydrogenase type 2 (*11βHSD2*) [3].

In teleosts, HPI axis activation commences only after hatch, while maternally derived cortisol plays an important role in early embryo development prior to de novo cortisol synthesis [4]. Egg-laying vertebrates transfer maternal hormones, including cortisol into the egg during vitellogenesis [6,7]. Consequently, maternal stress and the attendant elevation in cortisol levels may lead to abnormal embryo cortisol content in fishes and the associated developmental dysfunction and reduced offspring viability [8–12]. Recent studies have demonstrated that an abnormal increase in embryo cortisol levels causes developmental defects in zebrafish (*Danio rerio*) [4], including abnormal craniofacial phenotype [13] and cardiac defects [14].

Studies in viviparous animals have shown that fetal blood cortisol levels are tightly regulated to buffer the harmful effects of glucocorticoids on development [15]. The placental upregulation of *11βHSD2* by elevated maternal plasma cortisol levels is a mechanism protecting the fetus from the harmful effects of this steroid excess [16]. However, it is not known whether this excess stress steroid checkpoint exists in the ovarian follicles of oviparous animals. Most studies in fishes used exogenous cortisol manipulation of the embryos to mimic a maternal stress scenario [4,13,14]. No study has examined whether maternal stress will lead to excess cortisol deposition in the embryos of the asynchronous breeding zebrafish. Given the finding that excess zygotic cortisol levels influences developmental trajectories and phenotype in zebrafish [4], we used this species as a model to test the hypothesis that maternal stress leads to the transfers of excess cortisol to the embryos in this asynchronous breeder. This was tested by feeding mothers control or cortisol-spiked food for 5 days, followed by breeding daily for 10 days to measure temporal changes in embryo cortisol content. This breeding regimen was followed because the timing of oogenesis to ovulation in zebrafish is around 10 days [17]. We also carried out an *in vitro* study with isolated ovarian follicles from unstressed zebrafish mothers to determine whether elevated cortisol levels upregulate the gene encoding the enzyme *11βHSD2*, as a possible mechanism to limit the transfer of excess cortisol to the embryos during maternal stress.

## 2. Material and methods

### 2.1 Animal husbandry

Adult zebrafish (TL strain) were maintained in 10 l tanks on a recirculating system with a 14 : 10 light : dark cycle (Pentair Aquatic Habitats, Apopka, FL, USA). Water was maintained at 28°C, pH 7.6 and 750 μS conductivity. Animals were fed twice daily with Ziegler Adult Zebrafish Diet (Pentair Aquatic Habitats, Apopka, FL, USA).

### 2.2 Cortisol treatment

Twenty-four adult female zebrafish were assigned to each of two 10 l tanks. One group was fed control zebrafish diet as above, while the other group was fed diet laced with cortisol (25 μg cortisol (g body mass)^−1^ d^−1^). To date, no study has administered cortisol via feed to elevate endogenous cortisol levels in zebrafish; however, this is a commonly used technique in other teleosts to limit endogenous cortisol elevation due to handling of fish for injections [18]. As per these studies, zebrafish diet was prepared by soaking food pellets in 100% ethanol either alone (control) or with (0.5 mg per g feed) hydrocortisone (Sigma-Aldrich, St Louis, MO, USA) and allowing the ethanol to evaporate as previously described [18]. Fish were maintained in a recirculating system at a density of 3.5 g l^−1^ and were fed 2.5% of their body weight twice per day. The fish received an equivalent of 25 μg cortisol (g body mass)^−1^ d^−1^. We carried out preliminary studies to decide on the cortisol levels in the feed to mimic a maternal stress scenario. Fish were held under these conditions for 5 days, after which six fish from each group were sampled for measuring maternal cortisol levels. The remaining fish were transferred to breeding tanks, both groups were fed as in §2.1, and bred from the following day onwards for 10 days.

### 2.3 Fish sampling

On day 5, fish (*n*=6) from each group were euthanized with an overdose of 2-phenoxyethanol (1 : 1000 dilution; Sigma, Oakville, ON, USA). They were blotted dry and the body mass (BM), fork length (FL) and ovary weight (OW) recorded to calculate condition factor (*K*=BM×100/FL^3^) and gonadosomatic index (GSI=OW/BM×100). There were no significant differences in either the *K* or GSI between the two groups. Whole body and ovary were snap frozen on dry ice and stored frozen at −80°C for cortisol analysis later.

### 2.4 Breeding

Female fish from each treatment (*n*=18) were divided among six tanks (*n* = 3 per tank) along with two unexposed males in each tank. These tanks were divided into two breeding sets of three tanks for each treatment. Each breeding set was bred every other day for a total of 5 spawnings per set over a 10-day period. This approach was taken to avoid fatigue, and to maximize the daily embryo yield. For daily breeding set-up (at 18.00 h), fish were quickly netted and transferred into breeding traps in the same tank, and eggs were collected within 1 h of light (at 08.00 h) the following morning. Embryos were counted and pools of 20 were snap frozen on dry ice and stored at −80°C for cortisol analysis.

### 2.5 Cortisol analysis

Frozen whole body was ground into a powder with a chilled mortar and pestle on dry ice and extracted with diethyl ether (1 : 5 w/v) as described previously [19]. Embryos were not extracted, as preliminary studies revealed no difference in the cortisol content between the extracted and unextracted embryos. A competitive ELISA, that was validated for zebrafish, was used to quantify cortisol levels in embryos and mothers as described previously [20], with modifications. Briefly, high binding 96-well plates (Immulon HB, VWR) were coated with 100 μl of cortisol monoclonal antibody (1.6 μg ml^−1^; East Coast Bio, ME, USA) in phosphate buffered saline (1×PBS; 10× stock: 1.37 M NaCl; 27 M KCl, 18 mM KH_2_PO_4_; Na_2_HPO_4_) for 16 h at 4°C. The plate was then washed with PBS with 0.05% Tween 20 (300 μl well^−1^), and blocked with 0.1% bovine serum albumin (300 μl well^−1^; Sigma-Aldrich) for 1 h at room temperature. Standards comprised of hydrocortisone (Sigma) serially diluted (0–25 ng ml^−1^) in PBS and 50μl of either standards or samples were added to the wells in duplicate. Cortisol conjugated to horseradish peroxidase (1 : 1600 dilution; East Coast Bio, ME, USA) diluted in PBS was added to each well. Plates were incubated for 2 h, shaking, at room temperature. The plate was washed as described above, followed by the addition of the detection reagent (41 mM TMB and 8 mM TBABH in 200 mM potassium citrate, pH 4). After 25 min, the reaction was stopped with 1 M sulfuric acid and read at 450 nm using a microplate reader (VersaMax, Molecular Devices, CA, USA). The intra-assay and inter-assay coefficient of variation was 4.3% and 13.4%, respectively, with a minimum detection limit of 0.5 ng ml^−1^.

### 2.6 Cortisol regulation by ovarian follicles

Female fish (*n*=4) were euthanized with an overdose of 2-phenoxyethanol (1 : 1000 dilution; Sigma, Oakville, ON, USA). Intact ovaries were removed, and placed in a Petri dish of Leibovitz’s (L15) medium (Gibco, Grand Island, NY, USA). Ovarian follicles were mechanically dispersed using a Pasteur pipette and evenly divided into six wells of a 24-well plate. Follicles were immediately treated with cycloheximide (10 μg ml^−1^; Sigma-Aldrich, St Louis, MO, USA) or actinomycin D–mannitol (10 μg ml^−1^; Sigma-Aldrich) to block translation and transcription, respectively [21]. Cortisol (100 ng ml^−1^ hydrocortisone; Sigma-Aldrich) or vehicle control (0.01% ethanol) was added 30 min later and the follicles were incubated for 4 h at 28.5°C on a shaker. The follicles were centrifuged at 13 000*g* for 2 min, media removed, and the tissue stored at −80°C for transcript analysis.

### 2.7 Transcript abundance

Transcript levels of *11βHSD2* and *β*-actin in the ovarian follicles were measured by quantitative real-time PCR (qPCR) using gene specific primers as described previously [22,23]. Total RNA was extracted from ovarian follicles using Ribozol reagent (VWR, Mississauga, ON, USA) according to the manufacturer’s instructions, and quantified using a SpectraDrop Micro-Volume microplate (VersaMax, Molecular Devices, CA, USA). One microgram of RNA was treated with DNase I (Thermo Scientific, Waltham, MA, USA) to remove genomic contamination prior to cDNA synthesis using the High Capacity cDNA Reverse Transcription Kit (Applied Biosystems, Foster City, CA, USA) according to the manufacturer’s protocols. Transcript levels of *11βHSD2* and *β*-actin were measured by qPCR in triplicates using gene specific primers as described previously [23]. Briefly, 2 min at 94°C, 40 cycles of 95°C (30 s) and a primer specific anneal temperature (30 s; see below), followed by 10 min at 72°C. The *11βHSD2* forward primer (5^′^-TGCTGCTGGCTGTACTTCAC-3) and reverse primer (5^′^-TGCATCCAACTTCTTTGCTG-3^′^) had an annealing temperature of 55°C and an amplicon size of 123 bp. The *β*-actin forward primer (5^′^-TGTCCCTGTATGCCTCTGGT-3^′^) and reverse primer (5^′^-AAGTCCAGACGGAGGATGG-3^′^) amplified a 121 bp region at an annealing temperature of 60°C. A standard curve was used to determine relative transcript abundances according to established protocols [22]. All data were normalized to *β*-actin, as this transcript remained unchanged between treatments, and the data are expressed as per cent control.

### 2.8 Statistics

Data are shown as mean ± s.e. For overall embryo yield and cortisol levels (maternal, ovary, overall embryo), data were analysed by Student’s *t*-test. For temporal embryo cortisol levels, a two-way ANOVA was performed (Holm–Sidak *post hoc*). Significant interactions were tested using either a Student’s *t*-test (for treatment) or one-way ANOVA (for time effect). A repeated measure one-way ANOVA was performed for the ovarian follicle study (Holm–Sidak *post hoc*). Embryo cortisol levels were log transformed to meet the assumptions of normality and equal variance. Untransformed data are shown in all figures. A significance level (*α*) of 0.05 was used in all cases.

## 3. Results

### 3.1 Cortisol feeding

**Figure 1.**
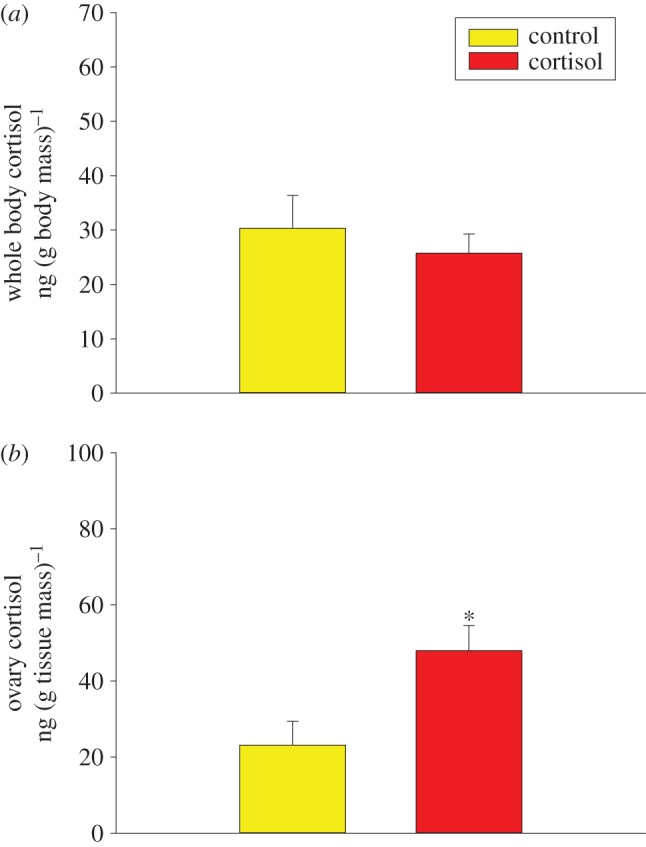
(*a*) Maternal cortisol levels. Whole-body cortisol content of adult female zebrafish following 5 days of feeding cortisol-laced food at a dose of 25 μg cortisol (g body mass)^−1^ d^−1^ (Student’s *t*-test, *t*=0.656,*p*= 0.527,*n*=6). (*b*) Maternal ovary cortisol levels. Ovary cortisol content of adult female zebrafish following 5 days of feeding cortisol-laced food. Values represent means ± s.e. Asterisks indicate significant effects (Student’s *t*-test, *t*=−2.718,*p*=0.0216,*n*=6).

**Figure 2.**
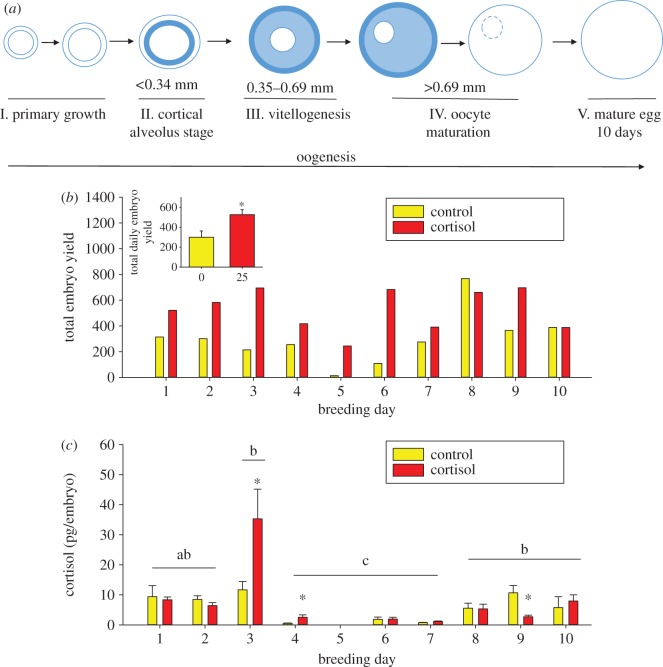
(*a*) Stages of follicle/oocyte development in zebrafish over 10 days (adapted from [17]). The primary growth phase (stage I), when follicles start to form and enlarge, is followed by the cortical alveolus stage (stage II), in which cortical alveoli accumulate within the oocyte. Stage III marks vitellogenesis, followed by oocyte maturation (stage IV). Stage IV is characterized by the migration of the germinal vesicle from the centre of the oocyte to the periphery, where the nuclear membrane is broken down. Stage V oocytes are ready for ovulation and spawning. (*b*) Total daily embryo yield. Embryos (1 h post-fertilization) were collected daily over a 10-day breeding period following 5 days of feeding cortisol-laced food (25 μg cortisol (g body mass)^−1^ d^−1^). Values represent the total number of embryos collected for each day of the 10-day breeding period. Inset: mean daily embryo yield. Values represent the mean ± s.e. of daily mean embryo yields over a 10-day breeding period. Asterisks indicate significant differences (Student’s *t*-test, *t*=−2.806,*p*=0.0117,*n*=10). (*c*) Mean daily embryo cortisol content. Embryos (1 h post-fertilization) were collected over a 10-day breeding period following 5 days of feeding cortisol-laced food (25 μg cortisol (g body mass)^−1^). Values represent the mean ± s.e. of cortisol values from embryos collected over a 10-day breeding period. Treatment effects within days are indicated by asterisks and overall time effects are indicated by different lowercase letters. A significant interaction was detected (two-way ANOVA, treatment × time: *F*=2.859,*p*=0.006, time: *F*=20.612,*p*<0.001, cortisol: *F*=3.474,*p*=0.065,*n*=5–10, Holm–Sidak *post hoc*). Day 5 has been omitted as insufficient embryos were yielded for cortisol analysis on this day.

There was no change in whole-body cortisol levels between control and cortisol-fed zebrafish (figure 1*a*, Student’s *t*-test, *t*=0.656,*p*=0.527). Cortisol content in the ovaries was twofold higher in the cortisol-treated fish compared with the controls (figure 1*b*, Student’s *t*-test, *t*=−2.718,*p*=0.0216). The proposed timing from oogenesis to spawning is around 10 days for zebrafish (figure 2*a*). The mean daily embryo yield over the 10 days was significantly higher in the cortisol group compared with the control group (figure 2*b*, Student’s *t*-test, *t*=−2.806,*p*=0.0117). There was no significant difference in the mean daily cortisol levels of embryos between the cortisol-fed and control groups (figure 2*c*, Student’s *t*-test, *t*=−0.366,*p*=0.719). Temporally, embryo cortisol content showed a significant time effect and interaction (figure 2*c*, two-way ANOVA, treatment × time: *F*=2.859,*p*=0.006, time: *F*=20.612,*p*<0.001, treatment: *F*=3.474,*p*=0.065). The cortisol levels of days 4, 6 and 7 embryos regardless of treatment were significantly lower compared with the other days. The days 3 and 4 embryos from the cortisol-fed mothers had significantly higher cortisol levels compared with the controls, while the day 9 embryos from the cortisol group had significantly lower cortisol levels compared with the control group. There was no embryo cortisol content on day 5 because the control group did not yield any embryos for comparison (figure 2*b*).

### 3.2 Ovarian follicle

**Figure 3.**
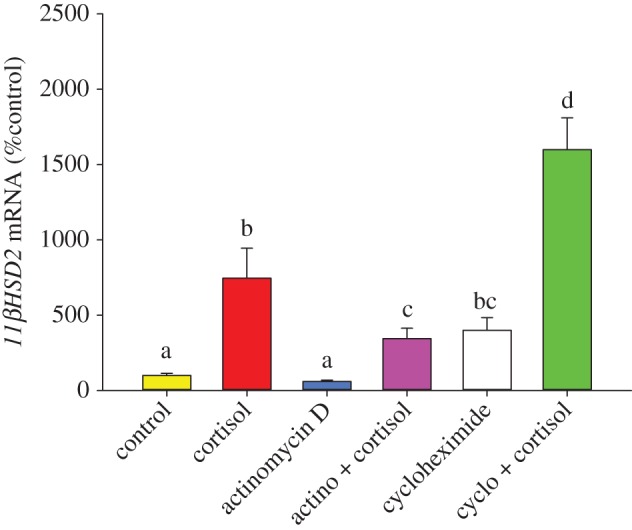
*11βHSD2* mRNA abundance in ovarian follicles. Follicles were treated with control (0.01% ethanol; yellow bar), cortisol (100 ng ml^−1^; red bar), actinomycin D (10 μg ml^−1^; blue bar), actinomycin D + cortisol (pink bar), cycloheximide (10 μg ml^−1^; white bar) and cycloheximide + cortisol (green bar) for 4 h in L15 media and *11βHSD2* mRNA levels measured. Data shown as mean ± s.e. (*n*=4 fish). Bars with different letters are significantly different (one-way repeated measures ANOVA, *F*=49.096<0.001, Holm–Sidak *post hoc*).

Transcript abundance of *11βHSD2* was sevenfold higher in cortisol (100 ng ml^−1^) treated follicles compared with vehicle (*p*<0.001;0.01% ethanol) treated control follicles (figure 3). Actinomycin D (10 μg ml^−1^) did not significantly alter *11βHSD2* transcript abundance (*p*=0.102). Actinomycin D in combination with cortisol resulted in threefold higher *11βHSD2* transcript levels compared with the control group (*p*<0.001; figure 3). However, this response was significantly lower compared with the cortisol-induced *11βHSD2* transcript level (*p*=0.039; figure 3). Cycloheximide treatment (10 μg ml^−1^) increased *11βHSD2* transcript abundance compared with the control group (*p*<0.001; figure 3). Combination of cycloheximide and cortisol-treated oocytes also had significantly higher transcript levels of this gene compared with either the cycloheximide (*p*<0.001) or cortisol-treated follicles (*p*=0.017; figure 3).

## 4. Discussion

Our results suggest that cortisol-mediated transcriptional upregulation of *11βHSD2* in ovarian follicles may be a key mechanism protecting embryos from elevated cortisol levels associated with maternal stress. We propose this tight control of embryo cortisol levels, despite the stress-induced elevation of this hormone in the mothers, as a key adaptive strategy to protect the embryo from the harmful effects of cortisol excess [4]. It has long been maintained that cortisol is transferred to the developing oocyte during vitellogenesis, and this hormone is essential for early development [4,6,7]. Indeed, de novo synthesis of cortisol commences only after hatch, and a cortisol hyporesponsive period during embryogenesis is essential for zebrafish development [4,23]. This is further justified by the developmental defects and poor survival seen in offspring raised from embryos with abnormal cortisol levels [4,13,14]. Consequently, it is essential that embryo cortisol levels are tightly regulated within narrow limits for proper development of zebrafish [4].

In the present study, feeding of cortisol-spiked food did not affect whole-body cortisol content. While the reason is unclear, this may be due to the 12 h time-lag between the last feeding and sampling for cortisol measurement. Also, it is important to note that we are measuring whole-body cortisol levels, and this may be masking any changes in the circulating levels of the steroid. This notion is supported by the higher cortisol content in the ovary of the steroid-treated fish, suggesting a clear compartmentalization in this target tissue and may reflect elevated circulating levels of this hormone. Another factor may be the elevated water temperature, as other cortisol feeding studies have used cool water fish [18]. Given the bolus route of cortisol administration, and the higher water temperature (28.5°C), it is likely that the steroid is cleared within 12 h in zebrafish. This is further supported by the observation that plasma cortisol clearance is faster in warm water fish compared with temperate fish [21], suggesting a key role for temperature in modulating the clearance rate of this hormone.

Our study is the first to report a temporal cortisol profile for embryos from the same asynchronous breeders over a 10-day period. The developing oocytes take around 10 days to progress from follicle to a mature egg in zebrafish (figure 2*a*) [17]. The yolk bodies begin to form in stage III oocytes (day 6) [24], and this is when steroid hormone incorporation is thought to occur [6]. Consequently, there would be a temporal delay (approx. 8–9 days) in the maximal cortisol content of freshly spawned embryos after the elevation in cortisol levels by feeding in the present study. The transient increase in embryo cortisol content seen only in embryos bred on day 3 and 4 after a 5-day cortisol feeding supports this temporal delay in cortisol accumulation in the oocytes. The result suggests that maternal stress-mediated cortisol deposition is not a passive process and actively regulated during oogenesis in zebrafish. Interestingly, the embryo cortisol levels in subsequent days (greater than 4 days) of breeding were similar in the cortisol and control groups (except on day 9), leading to the proposal that a control mechanism at the level of the ovarian follicles may regulate embryo cortisol levels in this asynchronous breeder. The temporal cortisol content even in the control group is highly variable. It is unclear if this reflects temporal differences in maternal cortisol content or whether this is a reflection of the frequency of breeding and warrants further study. To the best of our knowledge the variation in maternal cortisol deposition into the embryo and the associated mechanisms has yet to be studied in an asynchronous breeding fish.

Our result demonstrates for the first time that *11βHSD2* transcript levels in zebrafish ovarian follicles are upregulated in response to elevated cortisol stimulation *in vitro* (figure 3). *11βHSD2* is a key corticosteroid-silencing enzyme that catalyses the conversion of this steroid to its inactive metabolite cortisone [3]. This enzyme is active in the zebrafish ovary, including the theca and granulosa cells during early stages of oogenesis [25], and can be inhibited by 18*β*-glycyrrhetinic acid (18*β*-GA), a *11βHSD2* inhibitor [19], suggesting a key role for this enzyme in ovarian cortisol catabolism. Bioinformatics analysis revealed three putative glucocorticoid response elements in the promoter region of zebrafish *11βHSD2* [19], suggesting transcriptional regulation of this enzyme by cortisol in zebrafish follicles. In the present study, this was confirmed by a reduction in cortisol-induced *11βHSD2* transcript levels by the transcriptional inhibitor actinomycin D. Also, treatment with cycloheximide elevated the transcript levels in the cortisol group over and above that seen with just the steroid alone (figure 3). The elevation in *11βHSD2* transcripts in the cycloheximide group may include accumulation of basally expressed transcripts due to the translational blockade, along with newly transcribed mRNA in response to cortisol stimulation. Based on this result, we propose that stress-induced cortisol level increases *11βHSD2* mRNA turnover in zebrafish ovarian follicles leading to enhanced breakdown of this steroid to its inactive metabolite cortisone. The tight autoregulation of cortisol in the ovarian follicles due to upregulation of *11βHSD2* expression may be a key mechanism that limits the transfer of excess cortisol to the embryos from stressed mothers.

**Figure 4.**
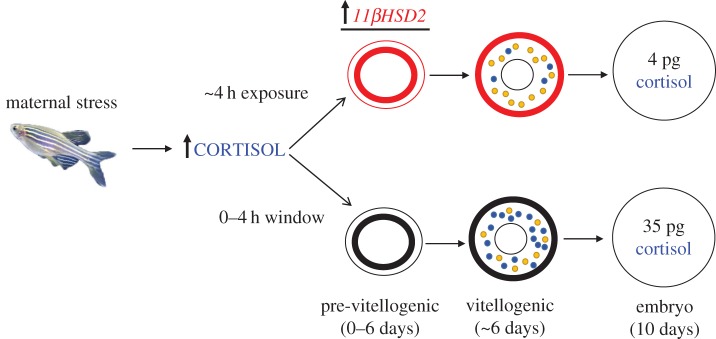
Working model for maternal deposition of cortisol into zebrafish oocytes. In oocyte development, cortisol (blue) is incorporated during vitellogenesis, along with vitellogenin (yellow). Therefore, excess cortisol may be deposited in the embryos in response to maternal stress and this is represented in the bottom panel of the figure; the cortisol content shown is based on the highest level observed in the breeding study (approx. 35 pg per embryo on day 3; figure 2*c*). Given that zebrafish are asynchronous breeders, oocytes are at multiple stages in the ovarian follicles and, therefore, pre-vitellogenic oocytes will also be exposed to cortisol excess during maternal stress. We propose these pre-vitellogenic oocytes will upregulate *11βHSD2* levels (by 4 h; figure 3) in the theca/granulosa cells surrounding the oocyte (top panel) in response to cortisol excess. This will result in the breakdown of cortisol to cortisone, thereby preventing excess maternal cortisol from being incorporated into the oocytes during subsequent vitellogenesis. This is reflected in the basal embryo cortisol levels (approx. 4 pg per embryo; figure 2*c*) seen in the cortisol group on days more than 4 days of breeding.

Based on the transient elevation in embryo cortisol content, our current working model is that oocytes undergoing vitellogenesis at the time of exposure to cortisol will incorporate this steroid into the oocytes of asynchronously breeding fish (figure 4). However, in response to exposure to cortisol excess, pre-vitellogenic follicles will upregulate *11βHSD2* in the theca/granulose cells and this may limit any further incorporation of excess cortisol into the oocytes during subsequent vitellogenesis (figure 4). We propose the observed transient rise in embryo cortisol content seen only on day 3 and 4 embryos, but not on subsequent days in the present study, may represent the steroid incorporation during a narrow window prior to the upregulation of *11βHSD2* by cortisol exposure. Studies are underway to test this *in vivo* by exposing either unstressed or stressed zebrafish mothers to *11βHSD2* inhibitor 18*β*-GA and measuring cortisol deposition in the oocytes and zygote. We propose that the function of *11βHSD2* in the zebrafish ovarian follicles corresponds to its role in the placenta of mammals, that is, protecting fetal tissue from exposure to elevated maternal cortisol levels [15,16]. Therefore, the ability of the ovarian follicles to regulate cortisol levels may be a highly conserved phenomenon in both the oviparous and viviparous vertebrates to limit the negative effects of cortisol on developmental programming events [4]. However, studies have shown a correlation between maternal stress/cortisol levels and elevated embryo cortisol content in other fishes [8–12], suggesting other factors, including species specificity, ovarian hormonal concentration and/or dysregulation of *11βHSD2*, that could potentially influence maternal transfer of this steroid.

An interesting finding from this study was that the fecundity was higher in the cortisol-treated mothers compared with the fed controls. Although this was not consistent daily, the majority of the days showed higher fecundity in the cortisol group. Increased fecundity has been shown previously in rainbow trout (*Oncorhynchus mykiss*) that were stressed early in vitellogenesis, compared with control fish or fish that were stressed late in oocyte maturation [26]. Most studies have focused on synchronously breeding salmonids, while little is known about stress/cortisol effects on asynchronous breeders [27]. Our results suggest that maternal stress, specifically increased cortisol stimulation, may act as a stimulus to increase spawning in the asynchronously breeding zebrafish. This cortisol-mediated increase in fecundity may be an adaptive strategy to ensure procreation during periods of stress. Indeed, there is evidence to support the hypothesis that cortisol may have a stimulatory effect on maturation processes [28,29], but the mechanism remains to be elucidated.

Overall, maternal stress and the associated elevation of cortisol levels may lead to transfer of excess cortisol to the embryos in the asynchronously breeding zebrafish. However, our results suggest that this elevated cortisol deposition is transient. We hypothesize that vitellogenic oocytes may be susceptible to excess steroid uptake, and this occurs only within a small window before the upregulation of *11βHSD2* activity in the ovarian follicles in response to higher cortisol content. This will result in the breakdown of cortisol to cortisone, thereby preventing any further steroid incorporation into the developing oocytes. We propose that this tight maternal control of oocyte cortisol deposition is an evolutionarily conserved phenomenon to effectively regulate and reduce excess cortisol incorporation in developing embryos, as excess zygotic cortisol content leads to developmental dysfunction [4].

## Data accessibility

Data available from the Dryad Digital Repository: http://dx.doi.org/10.5061/dryad.63bh4.
